# Quality assessment of peptide tandem mass spectra

**DOI:** 10.1186/1471-2105-9-S6-S13

**Published:** 2008-05-28

**Authors:** Fang-Xiang Wu, Pierre Gagné, Arnaud Droit, Guy G Poirier

**Affiliations:** 1 Department of Mechanical Engineering, University of Saskatchewan, 57 Campus Dr., Saskatoon, SK Canada, S7N 5A9, Canada; 2 Division of Biomedical Engineering, University of Saskatchewan, 57 Campus Dr., Saskatoon, SK Canada, S7N 5A9, Canada; 3 Health and Environment Unit, Eastern Quebec Proteomics Center, Laval University Medical Research Center (CHUL), Faculty of Medicine, 2705 Boul. Laurier, Ste-Foy, Quebec, Canada, G1V 4G2

## Abstract

**Background:**

Tandem mass spectrometry has emerged as a cornerstone of high throughput proteomic studies owing in part to various high throughput search engines which are used to interpret these tandem mass spectra. However, majority of experimental tandem mass spectra cannot be interpreted by any existing methods. There are many reasons why this happens. However, one of the most important reasons is that majority of experimental spectra are of too poor quality to be interpretable. It wastes time to interpret these "uninterpretable" spectra by any methods. On the other hand, some spectra of high quality are not able to get a score high enough to be interpreted by existing search engines because there are many similar peptides in the searched database. However, such spectra may be good enough to be interpreted by de novo methods or manually verifying methods. Therefore, it is worth in developing a method for assessing spectral quality, which can used for filtering the spectra of poor quality before any interpretation attempts or for finding the most potential candidates for de novo methods or manually verifying methods.

**Results:**

This paper develops a novel method to assess the quality of tandem mass spectra, which can eliminate majority of poor quality spectra while losing very minority of high quality spectra. First, a number of features are proposed to describe the quality of tandem mass spectra. The proposed method maps each tandem spectrum into a feature vector. Then Fisher linear discriminant analysis (FLDA) is employed to construct the classifier (the filter) which discriminates the high quality spectra from the poor quality ones. The proposed method has been tested on two tandem mass spectra datasets acquired by ion trap mass spectrometers.

**Conclusion:**

Computational experiments illustrate that the proposed method outperforms the existing ones. The proposed method is generic, and is expected to be applicable to assessing the quality of spectra acquired by instruments other than ion trap mass spectrometers.

## Background

High performance liquid chromatography (HPLC) coupled to tandem mass spectrometer provides an automated, high throughput approach widely used to generate peptide collision-induced dissociation (CID) mass spectra data for the analysis of complex biological protein mixture [1]. Most frequently, peptide identifications are made by searching CID mass spectra against a sequence database to find the best matching peptide in the database. From these assignments of spectra to peptides, the original proteins present in the sample are inferred. In addition, peptides corresponding to MS/MS spectra can be derived by de novo sequencing methods [2,3] and manually verifying methods. These methods are particularly useful in the case that search engines fail to assign high quality peptide CID spectra.

Over the past decade, many automated database search engines have been developed for assigning peptide CID mass spectra, for example SEQUEST [4], Mascot [5] and Sonar [6]. These search engines have successfully been applied to peptide CID mass spectrum assignments in many proteomics projects. However, majority of CID mass spectra in a proteomics project can often not be interpreted by any search engines, even after filtering poor quality spectra using some simple filters such as "most intensive peak selection" criterion [4-6]. For example, SEQUEST takes 138 hours to search 2*10905 MS/MS spectra, and finally 3404 spectra assignments are considered to be significant [7]. Supposed that searching each spectrum spends the same time, this example indicates that only 16% (= 3404/2*10905) of search time is useful while 84% of the search time (116 hours) is wasted. There are several reasons why the search engines failed to interpret the mass spectra. However, one of main reasons is that majority of MS/MS spectra are of too poor quality to be interpretable. In general, a spectrum is called to be of high quality if it is interpretable by a certain method, and otherwise it is called to be of poor quality. Even if high quality CID spectra that cannot be identified by any database search engine, they still are potential candidates to be interpretable for de novo sequencing or manually verifying method. Hence it is worthwhile to develop a powerful filter that masks out the poor quality of CID spectra before interpretation by any method for saving the time.

The earliest researches for assessing spectral quality have been reported from Yates Laboratory (The Scripps Research Institute). Tabb et al in this group [8] have assessed the spectral quality using a number of simple rules such as the minimum and maximum thresholds for the number of peaks, a minimum threshold on total peak intensity, and so on. They have claimed that such rules could remove 40% or more of the bad spectra. Later, Sadygove et al [7] for the same group have proposed a program called 2 to 3 to eliminate poor quality spectra and to narrow charge "uncertainty" – 2 to 3. The 2 to 3 method empirically determines the charge state of the precursor ion using its mass-to-charge ratio (*m*/*z*) and fragment ions contained in the tandem mass spectrum values. This approach has shown that by eliminating 20–30% of poor quality spectra, the search time required is decreased by 50%. The methods reported in these two papers just consider few features of spectra to describe the quality of spectra. Therefore it is not surprised that their filters perform poor.

Recently, some sophisticated methods have been reported for assessing spectral quality using more features of peptide CID mass spectra. The SPEQUAL proposed by Purvine et al. [9] uses three basic features: charge state, total signal intensity, and signal-to-noise estimates. Purvine et al. have claimed that the SPEQUAL may safely eliminate 55% of poor quality spectra. The three studies mentioned above do not give what percentage of high quality spectra is lost when the performances of their filters are achieved to eliminate their claimed percentage of poor quality spectra. Xu et al [10] have used four variables derived from five features of spectra to assess the spectra quality. These five features include three peak intensity-related features: the number of peaks larger than 5% of base peak intensity, 3% TIC (total ion current), and 2% TIC, and two peak distance-related features: the average peak distance along *m*/*z *for the peaks larger than 2% TIC, and the average distance along *m*/*z *for the peaks within 1.0~1.5% TIC. By trial and error, they have found a quadratic discriminant function which can be used to discriminate high quality spectra from high quality spectra.

More recently, Bern et al [11] used four of seven features of peptide CID mass spectra to establish the filters for singly charged spectra and multiply charged spectra, respectively. These seven features are Npeaks, Total Intensity, Good-Diff Fraction, Isotopes, Complements, Water Losses, and Intensity Balance. The best result reported in their paper [11] is that the method can remove 75% of poor quality spectra while losing 10% of the high quality spectra which are identifiable by SEQUEST. This paper develops a novel method for assessing the quality of peptide CID mass spectra, which can be used to establish a filter with the training data. Computational experiments on two datasets illustrate that the trained filters can eliminate majority the poor quality spectra while losing very minority of high quality spectra.

## Methods

### Properties of peptide predicted mass spectra

Many algorithms such as SEQUEST, Mascot and Sonar have been used to assign MS/MS spectra to peptides in a protein/peptide database. A key component of these algorithms is the score function, which evaluate the similarity between each experimental MS/MS spectrum and the predicted (theoretical) spectra of a given peptide in a database. A peptide in the database with the maximum similarity to the experimental spectrum is a likely candidate for the solution of the peptide identification. At this point, a perfect (highest quality) MS/MS spectrum is a peptide predicted spectrum theoretically produced according to the amino sequence of the peptide. In practice, no mass spectrometers can produce perfect MS/MS spectra. However, investigating the peptide predicted spectrum is extremely helpful in understanding the high quality spectra which could be potentially assigned to a peptide. Let *P *be a peptide consisting of *n *amino acids *a*_1_, *a*_2_,⋯, *a*_*n *_with respective masses *m*(*a*_*i*_). The mass of peptide *P *is calculated by

(1) $$m(P)=m(H)+m(OH)+\sum_{i=1}^{n}m(a_{i})$$

where *m*(*H*) and *m*(*OH*) are the additional masses of the peptide's N- and C- terminals. Hereafter, we will use *m*(*X*) to express the mass of molecule or a group of atoms *X*.

**Figure 1:**
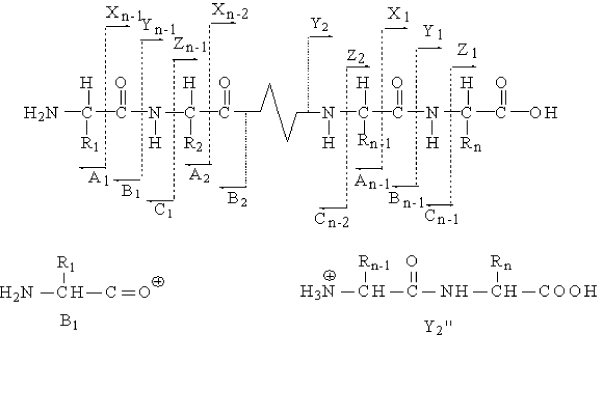
The schematic of the common peptide fragment ions.

From Figure 1, as peptide *P *fragments at backbone bond between the i-th and i+1-th amino acids counting from N-terminal, several types of ions could be produced in its predicted spectrum. The singly charged ion with N-terminal is denoted by $b_{i}^{+}$, and its m/z value is computed by

(2) $$m(b_{i}^{+})=m(H)+\sum_{j=1}^{i}m(a_{j})$$

The doubly charged ion with N-terminal is denoted by $b_{i}^{++}$, and its m/z value is computed by

(3) $$m(b_{i}^{++})=[m(b_{i}^{+})+m(H)]/2$$

The singly charged ion with C-terminal is denoted by $y_{n-i}^{+}$, and its m/z value is computed by

(4) $$m(y_{n-i}^{+})=2*m(H)+m(OH)+\sum_{j=i+1}^{n}m(a_{j})$$

The doubly charged ion with C-terminal is denoted by $y_{n-i}^{++}$, and its m/z value is computed by

(5) $$m(y_{n-i}^{++})=[m(y_{n-i}^{+})+m(H)]/2$$

From Equations (1) through (5) the following complementary equations

(6) $$m(P)+2*m(H)=m(b_{i}^{+})+m(y_{n-i}^{+})$$

(7a) $$m(P)/2+2*m(H)=m(b_{i}^{++})+(m(y_{n-i}^{+})+m(H))/2$$

(7b) $$m(P)/2+2*m(H)=(m(b_{i}^{+})+m(H))/2+m(y_{n-i}^{++})$$

(8) $$m(P)/2+2*m(H)=m(b_{i}^{++})+m(y_{n-i}^{++})$$

hold for a predicted (perfect) peptide spectrum. Therefore, equations (6) through (8) indicate that high quality spectra should have more complementary pairs of m/z values than poor quality spectra.

According to the CID fragmentation principle [12], these ions could lose a water or ammonia molecule. Therefore, high quality spectra should also have more pairs of m/z values with differences of a water molecular mass or an ammonia molecular mass for singly charged ions and with differences of half a water molecular mass or half an ammonia molecular mass for doubly charged ions than poor quality spectra. In addition, the N-terminal ions could lose a group CO while C-terminal could lose a NH group, Therefore, high quality spectra could have more pairs of m/z values with differences of (half) a CO mass or (half) an NH mass for (doubly) singly charged ions than poor quality spectra.

For a predicted spectrum, the difference between two consecutive singly charged N-terminal (C-terminal) ions is one of twenty amino acid mass weights. The difference between two consecutive doubly charged N-terminal (C-terminal) ions is half a mass weight of one of twenty amino acids. Therefore, high quality spectra should also have more pairs of m/z values with difference of (half) an amino acid mass weight for (doubly) singly charged ions than poor quality spectra.

### Features of peptide mass spectra

According to the properties of the predicted spectra, we introduce 12 discriminatory features to describe the quality of peptide CID mass spectra. These features may be classified into four categories: amino acid distances, complements, water or ammonia losses, and supportive ions. To do this, we first define four variables for a given peptide CID mass spectrum *S*_*E*_

(9) *dif*1(*x*, *y*) = *x *- *y*,   *x*, *y *∈ *S*_*E*_

(10) *dif*2(*x*, *y*) = *x *- (*y *+ 1)/2,   *x*, *y *∈ *S*_*E*_

(11) *sum*1(*x*, *y*) = *x *+ *y*,   *x*, *y *∈ *S*_*E*_

(12) *sum*2(*x*, *y*) = *x *+ (*y *+ 1)/2,   *x*, *y *∈ *S*_*E*_

#### (1) Amino acid distances

These features measure how likely two components in a peptide CID mass spectrum *S*_*E *_differ by one of twenty amino acids. Let

*DIF*_1 _= {(*x*, *y*)|*dif*1(*x*, *y*) ≈ *M*_*i*_, *i *= 1,⋯, 17}

*DIF*_2 _= {(*x*, *y*)|*dif*1(*x*, *y*) ≈ *M*_*i*_/2, *i *= 1,⋯, 17}

*DIF*_3 _= {(*x*, *y*)|*dif*2(*x*, *y*) ≈ *M*_*i*_,/2, *i *= 1,⋯, 17}

Where *M*_1_,⋯, *M*_17 _stand for the 17 mass weights of all 20 amino acids. In this study we consider all Methionine amino acids to be sulfoxidized and do not distinguish three pairs of amino acids in their mass: Isoleucine vs. Leucine, Glutamine vs. Lysine, and sulfoxidized Methionine vs. Phenylalanine as the masses of each pair are very close. The comparison implied by ≈ uses a tolerance which is set to 0.5 Thompson in this study, but can be changed by the user. The set DIF_1 _collects all pairs of singly charge ions in the spectrum *S*_*E *_that are different from one amino acid. The set DIF_2 _collects all pairs of doubly charged ions in the spectrum *S*_*E *_that are different from one amino acid. The set DIF_3 _collects all pairs of one doubly charged and the other singly charged ions that are different from one amino acid. Let

*F*_*i *_= |*DIF*_*i*_|,   *i *= 1, 2, 3

where |•| represents the cardinality of a set. If a tandem mass spectrum is produced from a peptide with well fragmenting, one expects that values *F*_*i *_(*i *= 1, 2, 3) calculated from this spectra should be much higher than those from a spectrum produced randomly.

#### (2) Complements

These features measure how likely an N-terminus ion and a C-terminus ion in the peptide CID mass spectra *S*_*E *_are produced as the peptide fragments at the same peptide bond. Let

*SUM*_1 _= {(*x*, *y*)|*sum *(*x*, *y*) ≈ *M*_*parent *_+ 2 * *m*(*H*)}

*SUM*_2 _= {(*x*, *y*)|*sum *(*x*, *y*) ≈ *M*_*parent*_/2 + 2 * *m*(*H*)}

*SUM*_1 _= {(*x*, *y*)|*sum *(*x*, *y*) ≈ *M*_*parent*_/2 + 2 * *m*(*H*)}

where *M*_*parent *_is the mass of precursor ion of the spectrum *S*_*E*_. The set *SUM*_1 _collects the complementary pairs of singly charged ions. The set *SUM*_2 _collects the complementary pairs of doubly charged ions. The set *SUM*_3 _collects the complementary pairs of one doubly charged ion and the other singly charged ion. For the same reason as the first three features, we define the other three features as the cardinalities of these three sets, i.e.

*F*_3+*i *_= |*SUM*_*i*_|,   *i *= 1, 2, 3

#### (3) Water or ammonia losses

These features measure how likely one ion in a peptide CID mass spectrum *S*_*E *_is produced by losing a water or ammonia molecule from other ion. Let

*WAD*_1 _= {(*x*, *y*)|*dif*1(*x*, *y*) ≈ *M*_*water *_*orM*_*ammonia*_}

*WAD*_2 _= {(*x*, *y*)|*dif*1(*x*, *y*) ≈ *M*_*water *_*orM*_*ammonia*_/2}

*WAD*_3 _= {(*x*, *y*)|*dif*1(*x*, *y*) ≈ *M*_*water *_*orM*_*ammonia*_/2}

Where *M*_*water *_and *M*_*ammonia *_are the mass of a water molecule and an ammonia molecule, respectively. The set *WAD*_1 _collects the pairs of singly charged ions with a difference of a water or ammonia molecule. The set *WAD*_2 _collects the pairs of doubly charged ions with a difference of a water or ammonia molecule. The set *WAD*_3 _collects the pairs of one doubly charged ion and the other singly charged ion with a difference of a water or ammonia molecule. Similarly, we define the next three features as the cardinalities of these three sets, i.e.

*F*_6+*i *_= |*WAD*_*i*_|,   *i *= 1, 2, 3

One can consider the water losses and the ammonia loss as separate features. This leads the resulting feature vector to have more components. In the classification problem, the more features do not mean leading a better classifier. The reverse state is often true as the more insignificant features could degrade the discriminatory power of other significant features.

#### (4) Supportive ions

These features measure how likely one ion in a peptide CID mass spectrum *S*_*E *_is a supportive ion. In this paper, we consider two kinds of supportive ions a-ions and z-ions. Although a-ions and x-ions are complementary if a peptide fragments at the specific bond shown in Figure 1, the a-ions are often generated by losing a CO group from b-ions [12], but not by fragmenting at the specific bond. With the same reason, we take z-ions into account but not c-ions

*AZD*_1 _= {(*x*, *y*)|*dif*1(*x*, *y*) ≈ *M*_*CO *_*orM*_*NH*_}

*AZD*_2 _= {(*x*, *y*)|*dif*1(*x*, *y*) ≈ *M*_*CO *_*orM*_*NH*_/2}

*AZD*_3 _= {(*x*, *y*)|*dif*2(*x*, *y*) ≈ *M*_*CO *_*orM*_*NH*_/2}

Where *M*_*CO *_and *M*_*NH *_are the mass of a CO group and an NH group, respectively. The set *AZD*_1 _collects the pairs of singly charged ions with a difference of a CO or NH group. The set *AZD*_2 _collects the pairs of doubly charged ions with a difference of a CO or NH group. The set *AZD*_3 _collects the pairs of one doubly charged ion and the other singly charged ion with a difference of a CO or NH group. Finally, we define the next three features as the cardinalities of these three sets, i.e.

*F*_9+*i *_= |*AZD*_*i*_|,   *i *= 1, 2, 3

At this point, we have introduced 12 features with physical meaning to describe the quality of peptide CID spectra. The four features *F*_*j *_(*j *= 1, 4, 7, 10) represent the evidence of existence of singly charged ions, called singly charged features. Although the other eight features have their own physical meaning, the pair of *F*_*k *_and *F*_*k*+1 _(*k *= 2, 5, 8, 11) could summed into one feature with a certain meaning. These four combined features represent the evidences of existence of doubly charged ions, called multiply charged features. As spectra with singly charged precursor ions are much different from those with multiply charged precursor ions, two separate classifiers should be trained: one for singly charged parent ions and one for multiply charged. The spectra with singly charge precursor ions are mapped into feature vectors with 4 singly charged features as it is impossible for the singly charged spectra to produce doubly charged ions. The spectra with doubly or triply charged precursor ions are mapped into feature vectors with all 12 features or feature vectors with 8 features which include four singly charged and four multiply features.

In principle, the high quality spectra are expected to have a number of feature values more than the poor quality spectra. On the other hand, the longer the peptide, the more the feature values have. This likely leads to a low sensitivity of the classifier as the high quality spectra produced from a shorter peptide would have a small number of feature values. To alleviate these effects, some normalization methods should be applied. In this study we tried two kinds of normalization methods: *F*_*i*_/*L*_*E *_and log(1 + *F*_*i*_)/log(*L*_*E*_), where *L*_*E *_is the estimated peptide length of a precursor ion. *L*_*E *_is computed by dividing the precursor ion mass by an average amino acid mass of 110 Daltons. The latter normalization method leads to better results, and thus has been adopted in this study.

### Classifier and validation

For our purpose, feature vectors of *n *peptide CID mass spectra has been summarized by an *n *× *p *matrix ***F ***= (*f*_*ij*_), where *F*_*ij *_denotes the value of feature *j *for peptide CID mass spectrum *i*. A classifier for determining spectral quality partitions a set of peptide CID mass spectra into two disjoint subsets, *A*_*H *_(for high quality spectra) and *A*_*P *_(for poor quality spectra), such that for a spectrum with feature vector ***f ***= (*f*_1_,⋯, *f*_*p*_) ∈ *A*_*x*_, the predicted group label is *X *(*X *= *H *or *P*). Classifiers are built from the spectra whose group labels are known. Such spectra with labels comprise the training set. In this study we has used Fisher linear discriminant analysis (FLDA) for building a classifier. FLDA is a classical method that models feature vectors of each group by multivariate Gaussian distributions. Although FLDA is assumed to analyze feature vectors subjected to multivariate Gaussian distributions, it is expected to perform well with summation features such as ours that have approximate Gaussian distributions due to the central limited theorem [13].

FLDA finds the discriminatory vector ***a ***such that the linear combinations ***fa ***of all feature vectors ***f ***= (*f*_1_,⋯, *fp*) have the maximum ratios of between-group to within-group sums of squares. Assume that an learning set consist of a set of feature vectors of high quality spectra *A*_*H *_and a set of feature vectors of poor quality spectra *A*_*P *_and each feature vector has *p *component. Let *n*_*x *_and ${\bar{f}}_{X}$ be the number of feature vectors and the mean vector of group *X *(= *H*, *P*), and $\bar{f}$ the mean of the whole learning set. It has proved that the discriminatory vector ***a ***is the vector which maximize ***a' Ba/a' Wa***, where ***B ***and ***W ***stand for the *p *× *p *matrices of between-group and within-group sums of squares and cross-products. Matrices ***B ***and ***W ***are calculated by

$$\begin{array}{c} B=\sum_{X=H,P}n_{X}({\bar{f}}_{X}-\bar{f})'({\bar{f}}_{X}-\bar{f})\\ W=\sum_{f\in A_{Hi}}\left(f-{\bar{f}}_{H})'(f-{\bar{f}}_{H}\right)+\sum_{f\in A_{Pi}}\left(f-{\bar{f}}_{P})'(f-{\bar{f}}_{P}\right) \end{array}$$

By Rayleigh's Quotient, the maximum values of ***a' Ba/a' Wa ***is the nonzero generalized eigenvalues of (***B***, ***W***), and the vector ***a ***is the corresponding generalized eigenvectors. The discriminant variable is defined as *u *= ***f****a*.

For a spectrum with feature vector ***f ***= (*f*_1_,⋯, *f*_*p*_), let $d_{X}(f)=\left|(f-{\bar{f}}_{X})a\right|$ denote the distance between the feature vector ***f ***= (*f*_1_,⋯, *f*_*p*_) and the mean ${\bar{f}}_{X}=({\bar{f}}_{X1},\cdots ,{\bar{f}}_{Xp})$ of group *X*, in terms of the discriminant variable, for the learning set. The predicted group label for the spectrum is the one whose mean vector ${\bar{f}}_{X}$ is closer to ***f ***in the space of the discriminant variable. That is, the spectrum is high quality according to FLDA if *d*(***f***) = *d*_*P*_(***f***) - *d*_*H*_(***f***) > 0, and otherwise it is poor quality. In this study, we use the following normalized value of *d*(***f***) to judge a spectrum,

$$nd(f)=\frac{d_{H}(f)-d_{P}(f)}{d_{H}(f)+d_{P}(f)}$$

Obviously, the function *nd*(***f***) has the range [1, -1], *nd*(***f***) = -1 if ${\bar{f}}_{H}=f$ while *nd*(***f***) = 1 if ${\bar{f}}_{P}=f$. For a given feature vector ***f ***of a spectrum, the larger the value of *nd*(***f***), the more likely the spectrum is of high quality. This study introduces a parameter *δ *to determine the quality of a spectrum. That is, a spectrum is considered to be of high quality if *nd*(***f***) > *δ*. The bigger the number *δ*, the more confident a spectrum is of high quality, and vice versa.

Once a classifier is well trained, it can be applied to predicting the group labels of each spectrum in the testing set. In the case that the group labels are known for the testing set, the predicted and true class label may be compared to estimate the correct rate of the classifier. In this study we calculate two correct rates: one for high quality dataset (denoted Rp) and one for poor quality dataset (denoted Rn)

$$\begin{array}{c} Rp=\frac{\text{\# of correctly predicted high quality spectra}}{\text{\# of high quality spectra}}\\ Rn=\frac{\text{\# of correctly predicted poor quality spectra}}{\text{\# of poor quality spectra}} \end{array}$$

In literature, Rp is also called the sensitivity while Rn the specificity. If there is just one labelled dataset at hand, two methods can be applied to evaluate the performance of a classifier. A simple estimate of the correct rates can be obtained by using the same learning dataset as the testing one. This method is commonly referred to as resubstitution. The correct rates resulting from resubstitution are called the apparent correct rates. For large datasets the apparent correct rates have only a small amount of bias for estimating the actual correct rates and can be used with little concern. Another method is called bootstrapping. The bootstrapping method can avoid the bias of correct rate estimates. In bootstrapping method, the dataset is randomly split into two disjoint datasets: one for learning and the other for testing, for a number of times. The average correct rates have little bias for large datasets. The bigger the value *δ*, the less the value Rp and the greater the value Rn. A good classifier is expected to have high values Rp and Rn with some value *δ*. In practice, some trade-off must be made between values Rp and Rn.

### Training datasets

#### TOV dataset

This dataset consists of 22,577 peptide CID mass spectra which were acquired on a LCQ DECA XP ion trap (Thermo Electron Corp.) in Eastern Quebec Proteomic Center in Laval University Medical Research Center in Canada. The samples analyzed were generated by the tryptic digestion of a whole-cell lysate from the human malignant epithelial ovarian tumor cell-line TOV-112D [14]. These MS/MS spectra were searched against a subset of the Uniref100 database (release 1.2, ) containing 44,278 human protein sequences using SEQUEST. The assignments of 2197 spectra were validated by PeptideProphet [14,15] to be correct. The 2159 multiply charged spectra out of these 2197 interpreted spectra are labelled as "high" quality spectra while the 17987 multiply charged spectra out of other 20380 uninterpretable spectra are labelled as "poor" quality spectra in this study. As the number of singly charged spectra is too small for training a reasonable classifier, this study does not train a classifier for singly charged spectra in this dataset.

#### Standard protein mixture (SPM) dataset

This dataset consists of 16727 peptide CID tandem spectra which were acquired on an ion trap and were provided by Institute of Systems Biology (ISB, Seattle, USA). The samples analyzed were generated by the tryptic digestion of a control mixture of standard 18 proteins (not of human origin) [16]. The MS/MS spectra were searched using SEQUEST against a human protein database (extracted from ) appended with the sequences of the 18 standard proteins and other common contaminants (totally, 5395 protein sequences in the final database). The assignments of 2067 peptide to the spectra were determined to be correct and other 13650 spectra are uninterpretable. The 2018 multiply charged spectra out of these 2067 interpreted spectra are labeled as "high" quality spectra while the 13635 multiply charged spectra out of other 13650 uninterpretable spectra are labeled as "poor" quality spectra in this study. For the same reason as for TOV dataset, this study does not train a classifier for singly charged spectra in this dataset.

The peptide CID mass spectrum is often expressed by the peak list, i.e., *S *= {(*x*_*i*_, *h*_*i*_) || 1 ≤ *i *≤ *m*}, where (*x*_*i*_, *h*_*i*_) denotes the fragment ion *i *with m/z value *x*_*i *_and intensity *h*_*i*_. Peptide bonds are not chemically equivalent within protonated peptides, and proton retention varies between moieties after peptide bond cleavage. For example, an N-terminal proline is an easily fragmented bond, and large b-ions are often degraded to a_2_-ions [12]. As a result, dissimilar intensities of fragment ions and/or incomplete fragment ion series are usually observed in CID of protonated peptides. Although it would be rash to assume that the more intense peaks were more "evident" than the weaker ones, it is true that peaks with relatively small intensity are more likely to be random noise. Peptide CID mass spectra often tend to exhibit a peak at every Thompson and thus include many small crowded "grass" peaks. In practice, peak rich, over-crowded peptide CID spectra are too noisy while less crowded spectra with prominent peak intensity distribution are more likely produced from some peptides of proteins in the sample and thus are good quality. It becomes essential to select more informative peaks for assessing the quality of peptide CID spectra. Our method selects the N most intense peaks of a peptide CID mass spectrum as its representatives for quality assessment, where N is a user-selected integer number. With a small N, some informative peaks could be lost. Alternatively, with a large N, more noise peaks might be included although little more informative peaks are selected. Reasonable numbers for N are between 50 and 200 [4,5]. Furthermore, since ion intensities are the results of many unknown factors and are yet difficult to utilize for spectral quality assessment, this study does not take into account intensity values of ions selected. Thus the peptide CID mass spectra in this study are reduced into a set of m/z values, and denoted by *S*_*E*_.

## Results

The proposed method has been tested on two tandem mass spectra datasets acquired by ion trap mass spectrometers: TOV dataset and SPM dataset (see section "methods" for the details about these datasets). To obtain more robust predicators, we have removed those feature vectors considered as outliers. A feature vector is considered to be an outlier if the Mahalanobis distance between the feature vector and the mean feature vector of its class fall in the extreme distance 5%. We map each spectrum into three feature spaces: one with all 12 features, one with 8 features (four doubly and four singly charged), and one with 4 singly charged features only. Three classifiers have been trained in these three feature spaces. The resubstitution method and bootstrapping method are used to evaluate their performance in terms of the sensitivity (Rp) and specificity (Rn) (see section "methods" for their definition).

**Figure 2:**
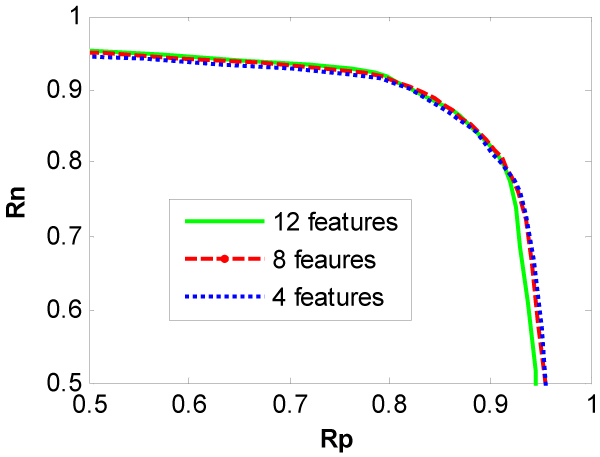
Plots of the correct rate Rn with respect to the correct rate Rp of classifiers for TOV dataset.

**Figure 3:**
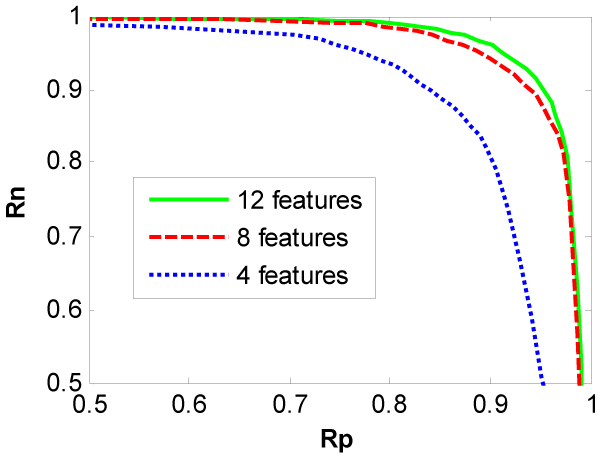
Plots of the correct rate Rn with respect to the correct rate Rp of classifiers for 18PM dataset.

In the bootstrapping method, the training dataset have been randomly split 20 times with the ratio of learning dataset size to testing dataset size: 80:20. The average correct rates Rp vs. Rn from the bootstrapping method are depicted in Figures 2 and 3 for TOV dataset and SPM dataset, respectively. For TOV dataset, the performance of three classifiers is comparable in terms of Rp and Rn. However, for SPM dataset, the performance of the classifiers with 12 and 8 features are comparable and clearly better than that with 4 features.

The results from the resubstitution method are almost the same as those from the bootstrapping method and are not depicted here due to the limitation of pages. Some special values of Rn and Rp from two methods are listed in Table 1 for both datasets to compare our classifier to the one proposed by Bern et al [11]. Bern et al [11] have claimed that the best of their classifiers can eliminate over 75% of the uninterpretable spectra while losing only 10% of the interpretable spectra. For TOV dataset, Table 1 shows that our classifier can eliminate over 83% of the uninterpretable spectra while losing only 10% of the interpreted spectra. Alternatively, it loses only 8% the identifiable spectra while eliminating 75% of the uninterpretable spectra. Table 1 also shows that our classifier performs better on SPM dataset than the one proposed by Bern et al. For this dataset, our classifier can eliminate over 97% of the uninterpretable spectra while losing only 10% of the interpreted spectra. Alternatively, it loses only 2% the interpretable spectra while eliminating 75% of the uninterpretable spectra. These results show that our method outperforms the existing methods.

**Table 1:** Some special rates of classifier for training datasets.

	TOV data		18 PM data	
	
	Rp (%)	Rn (%)	Rp (%)	Rn (%)
Resubstitution	90	83	90	97
Bootstrapping	90	83	90	96
Resubstitution	92	75	98	75
Bootstrapping	92	75	98	75

The developed classifier has been run on Dell Workstation PW350 to predict the quality of spectra. The workstation was equipped with Pentium 4/2.80 GHz processors, 512 MB RAM, and 75 GB hard drive. The operating system was Microsoft Windows 2000 Profession version. The developed classifier takes 245 seconds to process 20,000 spectra (or 0.0125 seconds per spectrum). SEQUEST takes 15 seconds per spectrum on a large (100 MB) database. Note that typically 16% spectra can be interpretable in a whole spectral dataset. This means that about 84% of search time may be save if the classifier is applied to filtering out the poor quality spectra before the SEQUEST search is applied to the original spectrum dataset.

## Conclusion

In this paper, we have done an initial research on assessing the quality of peptide CID spectra produced by tandem mass spectrometry. We have proposed 12 features to describe the quality of peptide CID mass spectra based on the properties of peptide predicted spectra (perfect spectra). Each spectrum has been mapped into feature vectors. We have employed the Fisher Linear discriminant analysis (FLDA) to construct the classifier in the feature space which distinguishes the high quality from the poor quality of peptide CID mass spectra. The proposed method has been tested on two tandem mass spectral datasets acquired by quadrupole ion trap mass spectrometers. Computational experiments have illustrated that the classifier developed in this study for assessing spectral quality is better than the one proposed by Bern et al [11] in terms of sensitivity and specificity.

The proposed method is generic for assessing the quality of peptide CID mass spectra although we has only tested on spectra produced by quadrupole ion trap mass spectrometers. One direction of the future work is to apply the proposed method to spectra produced by quadrupole time-of-flight mass spectrometers. Although the classifier is trained and tested on two dataset, to be practical the resultant classifier should be good at classifying other spectral datasets, at least those produced by the same type of instruments. The other direction of the future work is to test the classifier trained from one dataset on another dataset produced by the same type of instruments. In addition, incorporating the properties of the instrument which produces spectra into the feature vectors is expected to improve the performance of the classifier.

In this study, we consider spectra to be of high quality if they were significantly assigned to peptides by SEQUEST-based PeptideProphet software, and otherwise consider spectra to be of poor quality. A recent study [17] has shown that search results from the different search engines are very different and have few in common. Intuitively, spectra significantly assigned to peptides by any search engine should be of high quality. Therefore, an additional direction of the future work is to define a training spectral dataset with the results from as many search engines as possible, and then train a classifier on such datasets. It is expected that such a trained classifier is more reasonable than the one only based on the results from one search engine.

## Competing interests

The authors declare that they have no competing interests.

## Authors' contributions

FXW proposed the idea of this paper, implemented the programs, and drafted the manuscripts. PG, AD, and GGP carried out the collection of TOV data, participated in interpretation of data analysis results. PG helped to modify the manuscript. GGP conceived of the study and helped to modify the manuscript. All authors read and approved the final manuscript.
